# Epitachophoresis is a novel versatile total nucleic acid extraction method

**DOI:** 10.1038/s41598-021-02214-1

**Published:** 2021-11-23

**Authors:** Vladimira Datinska, Pantea Gheibi, Keynttisha Jefferson, Jaeyoung Yang, Sri Paladugu, Carolina Dallett, Ivona Voracova, Frantisek Foret, Yann Astier

**Affiliations:** 1 Roche Sequencing Solution, Pleasanton, CA 94588 USA; 2 Institute of Analytical Chemistry, Czech Academy of Sciences, 602 00 Brno, Czech Republic

**Keywords:** Biotechnology, Molecular biology, Oncology, Chemistry

## Abstract

Epitachophoresis is a novel next generation extraction system capable of isolating DNA and RNA simultaneously from clinically relevant samples. Here we build on the versatility of Epitachophoresis by extracting diverse nucleic acids ranging in lengths (20 nt–290 Kbp). The quality of extracted miRNA, mRNA and gDNA was assessed by downstream Next-Generation Sequencing.

## Introduction

Extraction of nucleic acids for molecular testing is a critical step in generating high quality sequencing libraries or input to PCR-based tests^1^. With the advent of multi-modality measurements for personalized medicine, clinical samples will be routed to many more molecular tests posing the pressure to improve every sample preparation step to derive insights^2^. The ideal extraction method extracts all intended analytes from a sample and enables automation. The conventional extraction systems such as spin columns and magnetic beads are commonly used for nucleic acids isolation from biological samples; however, these methods are labor intensive and difficult to automate^3^.

Non-affinity based separation methods such as electrophoresis, take advantage of differences in electro-migration to separate nucleic acids^4,5^. We designed and fabricated an easily automatable nucleic acids extraction system based on electrophoresis principles^6^. Epitachophoresis (ETP) takes advantage of a discontinuous electrolyte system consisting of leading and trailing electrolytes to focus nucleic acids into a narrow band^7,8^. In the ETP system, an agarose gel stabilizes the electrolyte solutions while allowing for nucleic acid size selection applications. When applicable, the gel can be replaced by a non-sieving matrix^9^ to allow for concentration of large DNA fragments.

**Figure 1:**
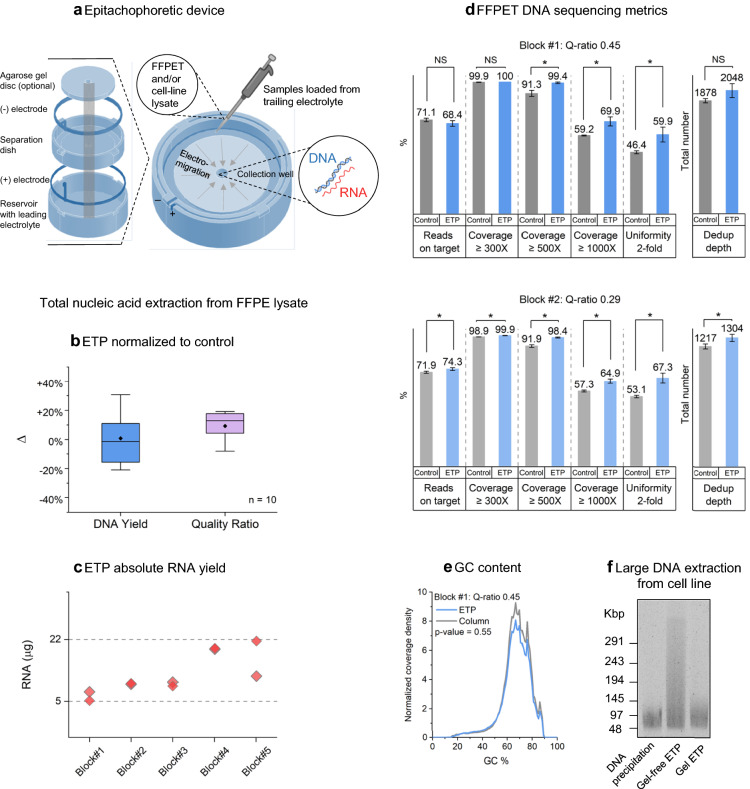
Epitachophoresis total nucleic acid extraction. (**a**) Exploded view of ETP device components. ETP device schematic, sample lysate injection and extract collection indicated. (**b**, **c**) Total nucleic acid extraction from FFPE tissue lysate. Two extraction replicas per method for five CRC FFPE blocks (n = 10). (**b**) Box plots of ETP DNA normalized to FFPE DNA column clean-up. Blue box—Qubit yield. Purple box—qPCR Quality ratio. (**c**) Scatter plot of ETP RNA Qubit yield (n = 10). (**d**, **e**) FFPE DNA Sequencing using AVENIO Tumor Tissue Targeted Kit. FFPE DNA column clean-up kit extracts were used as control to ETP extracted DNA. (**d**) Reads mapped on Target, Coverage (300X, 500X, 1000x), Uniformity, Total dedup depth. Quality block #1 Q-ratio 0.45 and quality block #2 Q-ratio = 0.29 (n = 4). (**e**) GC content of FFPE sequenced libraries (ETP—blue line, Control—grey line). (**f**) Pulse field gel electrophoresis of DNA extract from Lung Adenocarcinoma cell line (NCI-H1975). Line 1—Wizard^®^ Genomic DNA Purification Kit, Line 2 Gel-free ETP, Line 3 Gel ETP.

ETP utilizes a unique circular architecture where the sample is loaded into the outer ring and pipetted out as a liquid extract from the central well (Fig. 1a, Supplementary Fig. S1). We previously published a paper on ETP where we showed that we can extract and concentrate a DNA ladder loaded into 15 ml of trailing electrolyte^6^. Here we employed a challenging clinical sample, Formalin fixed paraffin embedded (FFPE)^10^ tissue, and a widely used biological model (cell line) to showcase the strength of ETP in extracting different types and sizes of nucleic acids from biological samples.

## Results and discussion

We applied ETP to the FFPE tissue samples. Total nucleic acid was extracted from colorectal (CRC) FFPE samples (n = 10, 5 blocks, 2 replicas per block) using the ETP platform, and DNA FFPE column extraction kit (Promega) was used as the control (Fig. 1b–e). ETP extracts had a similar DNA yield; mean yield value of ETP was 0.7% higher compared to control (Fig. 1b). To assess quality we relied on Q-ratio, which refers to the proportion of 191 bp fragments compared to the 66 bp fragments determined by qPCR measurement^11^ (“Methods” section). ETP extracts had superior Q-ratio; mean Q-ratio of ETP was 9.2% higher than control. Thus, the higher Q-ratio indicates that ETP extracted longer DNA fragments than control. Absolute ETP RNA yields were measured by Qubit (after DNAse treatment) from the same extracts (Fig. 1c). The high RNA yields (> 5 µg) highlighted the ability of ETP in extracting sufficient amount of both types of nucleic acids in a single run for sensitive downstream assays.

Sequencing library preparation of ETP extraction yielded superior results for different quality FFPE blocks. Two FFPE blocks of different quality were chosen for replicate DNA sequencing analysis^12–14^ (Fig. 1d). Normalized extracted nucleic acid inputs were used for sequencing library preparation (“Methods” section). We confirmed no significant GC content difference between ETP and standard column extraction (Fig. 1e, Supplementary Fig. S3). For block 1 (Q-ratio = 0.45), AVENIO DNA targeted enrichment libraries from ETP extraction had similar dedup-depth and on-target rates and superior somatic targets coverage (500x and 1000x), a highly desirable feature. Whereas, in block 2 (Q-ratio = 0.29), all the DNA sequencing metrics were better for ETP samples compared to control. In summary, ETP was capable of extracting high quality DNA from a low quality material.

We also looked into ETP’s ability to extract large molecular weight DNA. Gel ETP and a non-sieving (gel-free) ETP condition were used to extract DNA from lung carcinoma cell line (NCI-H1975). A DNA precipitation kit (Promega Wizard HMW DNA) was used as a reference. Pulsed-field gel electrophoresis was employed to estimate the size of the extracted fragments (Fig. 1f). The gel-free ETP resulted in the highest observed fragment size of above 290 Kbp and DNA precipitation method resulting in fragment sizes 48–145 Kbp.

**Figure 2:**
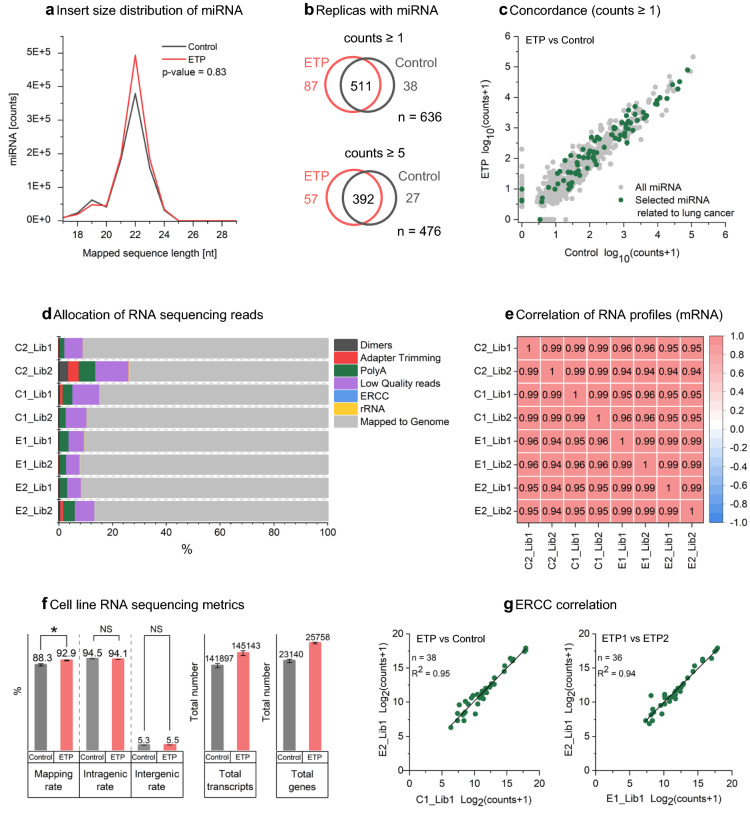
Lung Adenocarcinoma cell line ETP miRNA and RNA sequencing results from total nucleic acid extraction. (**a**–**c**) miRNA extraction and sequencing. Control—ReliaPrep™ miRNA Cell and Tissue Miniprep System kit. Sequencing libraries were subsampled to 20 million read pairs. (**a**) Fragment length distribution of mapped miRNA reads. ETP—plotted in red, Control—plotted in grey. (**b**) Venn diagram of miRNA detected from ETP and Control. Transcripts that are present in all samples ETP and column with normalized counts for each known miRNA ≥ 1, and ≥ 5. (**c**) Detection of miRNA implicated in lung cancer^15^. (**d**–**g**) RNA sequencing using random priming RNA sequencing library prep (Kapa RiboErase kit). Reads were subsampled to 20 million read pairs. Sample labeling as follows: ETP (E), Control (C) technical replicates (C1, C2, E1, E2) and Library replicates (Lib1, Lib2). (**d**) Allocation of reads. From left to right—filtered reads (primer-dimers, adapter dimers, PolyA trimming (< 30 nt), low quality reads (< 30 nt, Cut adapt quality = 28), reads aligned to ERCC, rRNA and mapped to the human genome. (**e**) Spearman correlation matrix for cell line RNA sequencing. Pairwise correlation score across ETP and column samples. Correlation scores range from − 1 (anti-correlation, blue) to 1 (strong correlation, red). (**f**) RNA Sequencing Metrics: Mapping rate, Intragenic (exons) and Intergenic (introns) rates, total detected transcript isoforms and genes. (**g**) ERCC Correlation between ETP and Control.

In addition to large DNA extractions, ETP technology can be also employed for small RNA (miRNA) applications. The sequencing analysis for miRNA extracted from lung carcinoma cell line (NCI-H1975) is presented in Fig. 2a–c and Supplementary Fig. S4. The average length of sequences mapped to mature miRNAs was between 20 and 25 nt (Fig. 2a), which matches the typical length of miRNAs reported in the literature^16^. The Venn diagram shows the number of unique and overlapping miRNA transcripts in ETP and control samples (Fig. 2b). For threshold count ≥ 1, we found 511 miRNA transcripts in both methods while ETP had 87 unique transcripts in comparison to control, which had 38. When a higher threshold is applied (counts ≥ 5), both methods extracted 392 transcripts in common while the unique transcripts for ETP were reduced to 57 and control to 27. In spite of the higher threshold and normalized library input, ETP was able to enrich more unique miRNA transcripts than control. The miRNAs related to lung cancer^15^ were detected and highlighted in green (Fig. 2c) compared to all found miRNAs (grey). The average of detected miRNA across 6 replicas was graphed. Zero counts were assigned to miRNAs not found in all replicates even if they were found in some of the libraries (dropouts from Fig. 2b). ETP is concordant to control and detected NSCLC miRNA across multiple logs.

We also focus on the total RNA profiles extracted from the lung carcinoma cell line (NCI-H1975) in addition to the targeted miRNA extractions. Pairwise comparisons of RNA from simultaneous RNA/DNA ETP (modified protocol in “Methods” section) and control replicates were correlated (Fig. 2d–g, Supplementary Fig. S5–S8). There is a consistent allocation of reads (Fig. 2d) from the same preparation protocol and strong positive correlation (> 0.9) in RNA expression between ETP and column extracted samples (Fig. 2e). The high Intragenic (94%) and low Intergenic (5%) rates for both extraction methods confirmed successful RNA library preparation and sequencing (Fig. 2f). The mapping rate, the percentage of all reads aligned to human genome reference, was 5% higher for ETP. Notably, ETP found 3246 more unique transcripts and 2618 more genes compared to the RNA column clean-up kit. Finally in our RNA experiments, we spiked-in prior to the extraction a synthetic ERCC RNA standard (various RNA lengths and concentrations, n = 92) in order to test ETP’s extraction range (Fig. 2g, Supplementary Fig. S7). Correlation plots between ETP replicates and ETP versus control were strongly correlated (R^2^ = 0.95).

## Conclusion

Through various quality control measurements and sensitive downstream Next-Generation Sequencing analysis, we confirmed quality and quantity of ETP extracted nucleic acids to be similar or better compared to conventional methods. From the same FFPE extracts, we obtained both DNA and RNA from a single run. From cell line extraction, we showed extraction of wide range of nucleic acids sizes—total RNA (miRNA, mRNA, protein-coding RNA, regulatory non-coding RNAs) and gDNA including large molecular weight. DNA and RNA quantity and quality was sufficient for Next-Generation Sequencing analysis.

## Methods

### List of chemicals, biological samples and kits

The following chemicals were used: l-histidine monohydrochloride monohydrate (99%), l-histidine (99%), *N*-tris(hydroxymethyl)methyl-3-aminopropanesulfonic acid (TAPS, 99.5%), tris(hydroxymethyl) aminomethane (TRIS, 99.8%), anionic dye Brilliant Blue FCF (Sigma-Aldrich). Low-electroendosmosis—multi purpose and molecular biology grade agarose (Fisher). DNA and RNA ladders (New England BioLabs). ERCC RNA Spike-In Mix (Thermo Fisher Scientific). SYBR Gold (Invitrogen).

Biological samples: Colorectal Cancer FFPE tissue blocks were purchased from Indivumed Germany (Hamburg, Germany) and curls were cut in-house with a microtome instrument (Leica, RM2255). NCI-H975 Lung carcinoma cell line was purchased from ATCC (Manassas, USA) catalog number of CRL-5908 and Lot number: 70023980.

List of kits used for sample preparation and extraction comparison: All following kits were purchased from Promega: ReliaPrep FFPE gDNA Miniprep System, ReliaPrep FFPE Total RNA Miniprep System, Wizard HMW DNA Extraction Kit, ReliaPrep miRNA Cell and Tissue Miniprep System, ReliaPrep RNA Tissue Miniprep System, ReliaPrep gDNA Tissue Miniprep.

List of analytical kits used in the presented study: Qubit assay kits (Thermo Fisher Scientific)—1x dsDNA HS Assay Kit, RNA HS Assay Kits, microRNA Assay Kit. Bioanalyzer kits (Agilent)—High Sensitivity DNA Kit and High Sensitivity RNA kit. Tapestation kits (Agilent)—High Sensitivity DNA ScreenTape, Genomic DNA ScreenTape, High Sensitivity RNA ScreenTape.

List of kits used for sequencing libraries: Amicon Ultra-0.5 mL filters (3 and 10 KDa) were purchased from Sigma Millipore. Sequencing libraries for FFPE DNA extraction were prepared using the AVENIO Tumor Tissue Expanded kit Analysis workflow (Roche Diagnostics, for Research Use Only. Not for use in diagnostic procedures). miRNA libraries were prepared using NebNext small RNA preparation for Illumina (set 1, New England BioLabs). Sequencing libraries for total RNA were prepared using the KAPA RNA HyperPrep Kit with RiboErase (Roche Diagnostics, for Research Use Only. Not for use in diagnostic procedures).

### ETP device and equipment

Fabrication of ETP device: We used Autocad (Autodesk) 3D modeling software for designing the platform (Fig. 1a). The final polypropylene ETP device with outer dimensions of 100 × 100 × 30 mm was fabricated by injection molding (Fathom manufacturing, Oakland, USA). The ETP device was equipped with electrodes. Cathode (90 mm OD, 5 mm height) was fabricated from stainless steel (D&V Precision Sheet Metal, Indianapolis, USA) and is inserted in the top dish (90 mm ID, 20 mm depth). The bottom dish anode (5 mm height) was laser-cut from MinGraph Flexible Graphite Sheet with Adhesive. It was gently taped around the perimeter of the bottom dish (100 mm ID, 30 mm depth). A plastic cup with a semipermeable membrane: Slide-A-Lyzer™ MINI Dialysis Units 2000 Da MWCO (Thermo Fisher Scientific) was inserted into the central collection well equipped with 1.2 × 9 mm o-ring (McMaster). To minimize the volume the Slide-A-Lyzer was cut in half by a razor blade creating a collection cup with a volume of less than 150 µl. Custom made polycarbonate mold for gel (75 mm OD and 5 mm ID barrier for central hole, 4 mm depth) was fabricated at AJ solution machining (Fremont, USA). Gels were covered by plastic lid (75 mm OD, 5 mm ID central hole) made by laser cutting from gel bond film (Lonza).

Equipment: PowerPac™ HC High-Current Power Supply was purchased from BioRad and uEP300-12 Power Supply was from Labsmith (Livermore, USA). Benchtop pH Meter Orion Star A211 with ROSS pH Buffer calibration kit were obtained from Thermo Scientific. Laser cutter—Speedy 360 with a 120 W CO^2^ power source was used (Trotec). Qubit 4 fluorometer (Thermo Fisher Scientific) was used for quantitative amounts of DNA and RNA from extracts. Size of extracted DNA and RNA was checked on Bioanalyzer 2100 (Agilent), Tapestation 4200 (Agilent) and Pulse Field Electrophoresis Pulse Pippin (Sage Science). Extracts were processed on Roche 480 lightCycler (Roche), Illumina NextSeq 500 (Illumina), Veriti Dx (Thermofisher).

### ETP separation conditions

Electrolytes and gel were prepared by following procedure: Leading electrolyte (LE) solution—100 mM Histidine-HCl, pH 6.25 (10.49 g of l-histidine monohydrochloride monohydrate 11 g of l-histidine in 500 ml water). Trailing electrolyte (TE) solution—20 mM TAPS-TRIS, pH 8.30 (0.605 g of TAPS and 1.625 g of TRIS in 500 ml of water). The agarose gel—in 20 mM LE (Histidine-HCl; pH 6.25). All buffers were prepared in deionized and nuclease-free water (Fisher). 0.5% (or 0.7%) agarose gel—500 mg (or 700 mg) of agarose was mixed with 100 ml of 20 mM Histidine-HCl LE buffer in a glass Erlenmeyer flask and heated on the hot plate till boiling while stirring. The mixture was kept at boiling for 1 min. After cooling the mixture to approximately 60 C, the solution was transferred to the round gel mold. Gel should be prepared at least 1 h before extraction and can be kept at 4 C for overnight. 0.7% agarose gel was used for FFPE experiments, 0.5–0.7% agarose gel was used for cell line experiments.

Device set-up:

- Top dish: the dialysis cup was inserted into the central well.
- The bottom dish was filled with 100 ml of LE.
- The top dish was inserted into the bottom dish.
- The central well was filled with LE to prevent air pockets.
- The gel was placed on the top dish.
- The gel was covered with the cover lid.
- The biological sample was mixed with 8 ml of TE and Brilliant blue (10 µl 0.1 mg/ml in water).
- The sample/TE mixture was injected into the gap between the gel and the electrode.
- Constant power 2 W was applied for 40 min.

Detection and extract collection: The Brilliant blue was used to indirectly track the nucleic acid band (Supplementary Fig. S1). Another contactless method of tracking the movement of ions was monitoring the change in voltage. Upon reaching a predetermined ideal voltage for each application (Supplementary Fig. S2), ETP was stopped and the extract was collected. The collection volume typically ranged from 150 to 180 µl. Due to the limitation of the volume input for the downstream assays, Amicon filter unit was utilized to reduce the volume.

### FFPE tissue (total nucleic acids extraction)

Sample pretreatment (Deparaffinization, lysing, Proteinase K treatment & de-crosslinking): Using Promega ReliaPrep FFPE gDNA Miniprep System, FFPE curls (2 × 10 µm curls per extraction) were deparaffinized, lysed and Proteinase K treated as described by the manufacturer. Lysates were then de-crosslinked at 80 C for 1 h before cold shock on ice for 2 min and stored at 4 C overnight. Samples were then thawed at room temperature for at least 10 min prior to extraction via ETP. This extraction type had two biological replicas for each FFPE block (5 CRC blocks: 10 extraction total).

Control extractions used for FFPE study: Promega ReliaPrep FFPE gDNA Miniprep system was used according to the manufacturer to extract DNA from FFPE curls (2 × 10 µm curls per extraction) with minor modification of RNase treatment step being omitted. This extraction type had two biological replicas for each FFPE block (5 CRC blocks: 10 extraction total).

Nucleic Acids yield measurement: In order to have true measurement of dsDNA and RNA without background interference, small portions of each extract underwent RNase A (Promega: A797C from FFPE gDNA kit) or DNase I (New England Biolabs: RNase-free, M0303S) treatments. RNase treated portion was used to measure the true dsDNA amount (Qubit dsDNA HS Assay Kits) and DNase treated samples were used to measure the accurate RNA amount (Qubit RNA HS Assay Kit).

FFPE DNA Quality measurement: DNA Quality was assessed using the AVENIO Tumor DNA Isolation and QC Kit (Roche Diagnostics). The quality of each FFPE DNA sample was assessed by quantitative PCR (qPCR) using 2 PCR amplicons (66 bp and 191 bp). The normalized quality ratio (Q-ratio) was calculated using QC PCR DNA standard of known quality. We used the 3 technical replicates of the Cp values per amplicon to calculate the Q score for each sample including the QC PCR DNA standard by using the following equation: Q score = 2[average(Cp66) − average(Cp191)]. We obtained a Q-ratio for each sample through the following equation: Q-ratio = sample Q score/QC PCR DNA Standard Q score.

FFPE DNA Library preparation & sequencing: Sequencing libraries for FFPE DNA extraction were prepared using the AVENIO Tumor Tissue Expanded kit Analysis workflow (Roche Diagnostics; for Research Use Only; Not for use in diagnostic procedures). Input was calculated (100/(Q-ratio) + 10) ng of input DNA where Q-ratio is the normalized quality score from qPCR assay. Libraries were sequenced using Illumina NextSeq 500 High Output v3 (300 cycles, Illumina). The sequencing data were analyzed using an internal analysis pipeline equivalent to the commercially available AVENIO Oncology Analysis Software version 2.0.0. Reads were subsampled to 20 million paired end reads. Two extraction replicas and two libraries per replica (n = 4) from CRC FFPE block for each extraction method.

### Cell line (miRNA)

Sample pretreatment (cell lysing and Proteinase K treatment) for ETP samples: 100 µl Lysis buffer and 10 µl Proteinase K from Promega ReliaPrep FFPE Total RNA Miniprep System kit was used to pretreat the ~ 325,000 cells per extraction. The mixture was digested at 56 C for 15 min. Lysates were then cold shocked on ice for 2 min before overnight incubation at 4 C. All samples were thawed at room temperature for at least 10 min prior to extraction.

Extracts post-treatment (DNase treatment + RNA Column Clean-up): Prior to downstream analysis, DNA portion of the extraction was digested by 20 min treatment with NEB DNase I (RNase Free) at 37 C. Total RNA was retrieved from the treated sample using NEB Monarch RNA Clean-up kit (10 µg) with 3X ethanol used alongside the binding buffer to ensure retention of the small RNA in the extracts (> 6 nt). This protocol was suggested by the manufacturer in keeping the small RNA during clean-up. This condition had three extraction replicas.

miRNA extraction control: The Promega ReliaPrep miRNA cell and Tissue miniprep System was used to pretreat and extract total RNA from chosen cell lines as recommended by the manufacturer. Extracts were then further concentrated by NEB Monarch RNA Clean-up (10 µg) kit prior to small RNA library preparations. Please note that conditions used for the RNA Column clean-up was tailored to keeping any RNA above 6 nt size (3X used for ethanol).

Nucleic Acids yield measurement: Qubit RNA HS assay kit (Thermo Fisher Scientific) was used to measure total RNA from each extraction while Qubit microRNA assay kit (Thermo Fisher Scientific) was employed to estimate the amount of small RNA (< 1000 nt) extracted per sample.

RNA size range measurement following extractions: RNA size was profile was checked by Tapestation capillary gel electrophoresis (High Sensitivity RNA ScreenTape for Tapestation 4200, Agilent) and by Bioanalyzer 2100 chip gel electrophoresis (High Sensitivity RNA, Agilent).

miRNA cell line Sequencing Analysis and data analysis: Total RNA was extracted from the (cell line). 500 ng was used as an input into the NebNext small RNA preparation for Illumina (set 1, New England BioLabs). Library preparation was performed according to manufacturing instructions. RNA extract was normalized 500 ng prior to generating the small RNA libraries. Ampure XP beads was used as the size selection method. Two rounds of bead clean-up was preformed. The purified libraries were pooled and then sequenced with a NextSeq 500 instrument using the Illumina NextSeq High Output kit v3 (300 cycles) 2x 75 (Illumina).

We have used the publicly available nextflow pipeline to process the miRNA sequencing data. https://github.com/nf-core/smrnaseq. The raw sequencing data and the normalized CPM (counts per million) values for each of the samples is available at: https://www.ncbi.nlm.nih.gov/geo/. Venn diagram input data: Only miRNA transcripts with abundance (greater than or equal to a predefined detection threshold) in all the ETP replicas or in all Column replicas are considered to be extracted by the respective method. Scatter plot: The average of detected miRNA across 6 replicas was graphed. Zero counts were assigned to miRNAs not found in all replicates even if they were found in some of the libraries (dropouts from Fig. 2b). All miRNA relevant to lung cancer are included^15^.

### Cell line (total nucleic acids extraction)

Sample pretreatment (cell lysing and Proteinase K treatment) for ETP: 100 µl Lysis buffer and 10 µl Proteinase K from Promega ReliaPrep FFPE Total RNA Miniprep System kit was used to pretreat the ~ 557,000 cells per extraction. Treatment was done at 56 C for 15 min. Lysates were then cold shocked on ice for 2 min prior to being incubated overnight at 4 C. Following overnight incubation, samples were first incubated at room temperature for at least 10 min prior to the next steps. ERCC was added into the cell lysate—1 µl of 1:100 dilution of ERCC RNA Spike-In Mix (Thermo Fisher Scientific). Lysates were fragmented into smaller pieces (500 bp) using Covaris E220 Focused UltraSonicator (Covaris, Woburn, USA). For the fragmentation step, 130 µl of lysate solution was placed into covaris microtubes and the following sonication program was used: duty cycle 10%, intensity 3, cycles per burst 200, time 80 s.

Control conditions: DNA Column Clean-up Control—Promega Wizard SV Genomic DNA Purification System was used according to the suggested protocol by the manufacturer to extract gDNA from ~ 557,000 cells per extraction. ERCC were added into the cell lysate prior to extraction of nucleic acids by the columns. This condition had two technical replicates.

RNA Column Clean-up Control: Promega ReliaPrep RNA cell Miniprep System was used according to the suggested protocol by the manufacturer to extract RNA from ~ 557,000 cells per extraction. ERCC were added into the cell lysate prior to extraction of nucleic acids by the columns. This condition had three technical replicates.

Nucleic Acids yield measurement: Qubit RNA HS assay kit and Qubit dsDNA HS assay kit were used to measure total RNA and dsDNA respectively from each extraction which was used to calculate the input for each sequencing Library preparations.

DNA & RNA size profile measurement: Size of profile extracted DNA and RNA was determined using Tapestation 4200 (Agilent) by Genomic DNA ScreenTape Analysis and High Sensitivity RNA ScreenTape (Agilent) respectively. For RNA, samples were first treated with DNase I (New England Biolabs: RNase-free, M0303S) and underwent RNA Column clean up before size profile analysis and the RNA sequencing library preparations to avoid any background signal from DNA.

Cell line RNA Library preparation & sequencing: Sequencing libraries for RNA extraction were prepared using the KAPA RNA HyperPrep Kit with RiboErase (Roche Diagnostics). Libraries were sequenced using Illumina NextSeq 500 High Output v3 (300 cycles, Illumina). The sequencing data were analyzed using an internal analysis pipeline equivalent to the commercially AVENIO Oncology Analysis Software (version 2.0.0). Reads were subsampled to 20 million paired end reads.

Cell line RNA Sequencing Analysis: We used HISAT2 to align the sequencing reads to the genome and then use Kallisto to quantify the abundance of RNA transcripts. Gene abundances were calculated by summing over the abundance of the corresponding transcripts for the gene.

Cell line DNA Library preparation & sequencing: Sequencing libraries for cell line DNA extraction were prepared using the AVENIO Tumor Tissue Expanded T Analysis workflow (Roche Diagnostics). Input in Library preparation with 100 ng. Libraries were sequenced using Illumina NextSeq 500 High Output v3 (300 cycles, Illumina). The sequencing data were analyzed using an internal analysis pipeline equivalent to the commercially AVENIO Oncology Analysis Software (version 2.0.0). Reads were subsampled to 20 million paired end reads. Two extraction replicas and two libraries per replica (n = 4) from CRC FFPE block for each extraction method.

### Cell line (long DNA extraction)

Sample pretreatment (cell lysing and Proteinase K treatment): Promega wizard HMW DNA extraction lysis buffer and Proteinase K were used as described by manufacturing to lyse ~ 290,000 cells per extraction. Wide bore pipette tips were used to reduce the DNA shearing. This condition had three extraction replicas. Following lysing and proteinase K treatment, lysate was loaded onto ETP and long DNA was extracted.

Gel ETP conditions were same as described in “ETP separation conditions” section above.

Gel-free ETP condition and set-up: A miniaturized version of the EPT device was used. The polycarbonate top dish (25 mm ID, 15 mm depth) was fabricated by injection molding (Protolab, USA). A 6-well plate served as the bottom dish (17.0 ml Multiwell Cell Culture Plates, VWR). The anode (3 mm height) was laser-cut from MinGraph Flexible Graphite Sheet with Adhesive. It was gently taped around the perimeter of the outside top dish. Stainless steel ring (OD 25 mm, 2 mm height, Smalley, USA) served as the top cathode electrode. Separation semipermeable membrane (10 × 30 mm) was cut from 0.45 µm Durapore Membrane Filter (Millipore, Sigma). The separation membrane was then carefully rolled around and attached to insert by using Double side adhesive tape (0.1 mm thick acrylic adhesive, McMaster). Inserts were CNC machined from polycarbonate (6 × 8 × 5 mm, heightxODxID). The insert equipped with a separation membrane and o-ring 1.9 × 5.7 mm was inserted to the top dish central hole (9 mm ID). Dialysis membrane (Acetate cellulose, 10 KDa MWCO) was attached by double side adhesive (M3) acrylic at the bottom of the top dish with insert.

ETP separation condition: Separation—8.3 ml of 100 mM LE in the bottom dish and 200 µl of 100 mM LE inside the central well. Sample: 0.9 ml of 20 mM TE buffer, Brilliant blue (10 µl 0.1 mg/ml in water) and 320 µl of lysate. Constant power 0.5 W was applied for 15 min.

Control condition/commercial kit for long DNA extraction: Wizard HMW DNA Extraction kit was used to extract long DNA from chosen cell line (~ 290,000 cells per extraction) with three extraction replicas for this condition. Protocol suggested by the manufacturer was followed with RNase treatment omitted. Wide orifice pipette tips (Rainin) were used to reduce the DNA shearing.

Gel-pulse field analysis: The Pulse field gel electrophoresis was run using the Pulse Pippin instrument (Sage Science). We used a 0.75% agarose gel (SeaKem GOLD, Lonza) in a 0.5X KBB buffer. Lambda PFG Ladder was used as a standard (New England Biolabs). The power supply was set for 5–430 Kbp Waveform protocol (75 V, 16 h). After electrophoresis, the gel was stained for 30 min in SYBR safe gel stain solution in 0.5X solution (Invitrogen). The pictures of the gel were imaged using UV light (Protein Simple).

## Supplementary Information

Supplementary Information.
