# Supplementary material for: Epitachophoresis is a novel versatile total nucleic acid extraction method
Source: Sci Rep. 2021 Nov 23;11:22736. doi: 10.1038/s41598-021-02214-1 (PMC8611068; doi:10.1038/s41598-021-02214-1)
Supplement: Supplementary file 1 — Supplementary Information. [file 41598_2021_2214_MOESM1_ESM.pptx]

## Slide 1
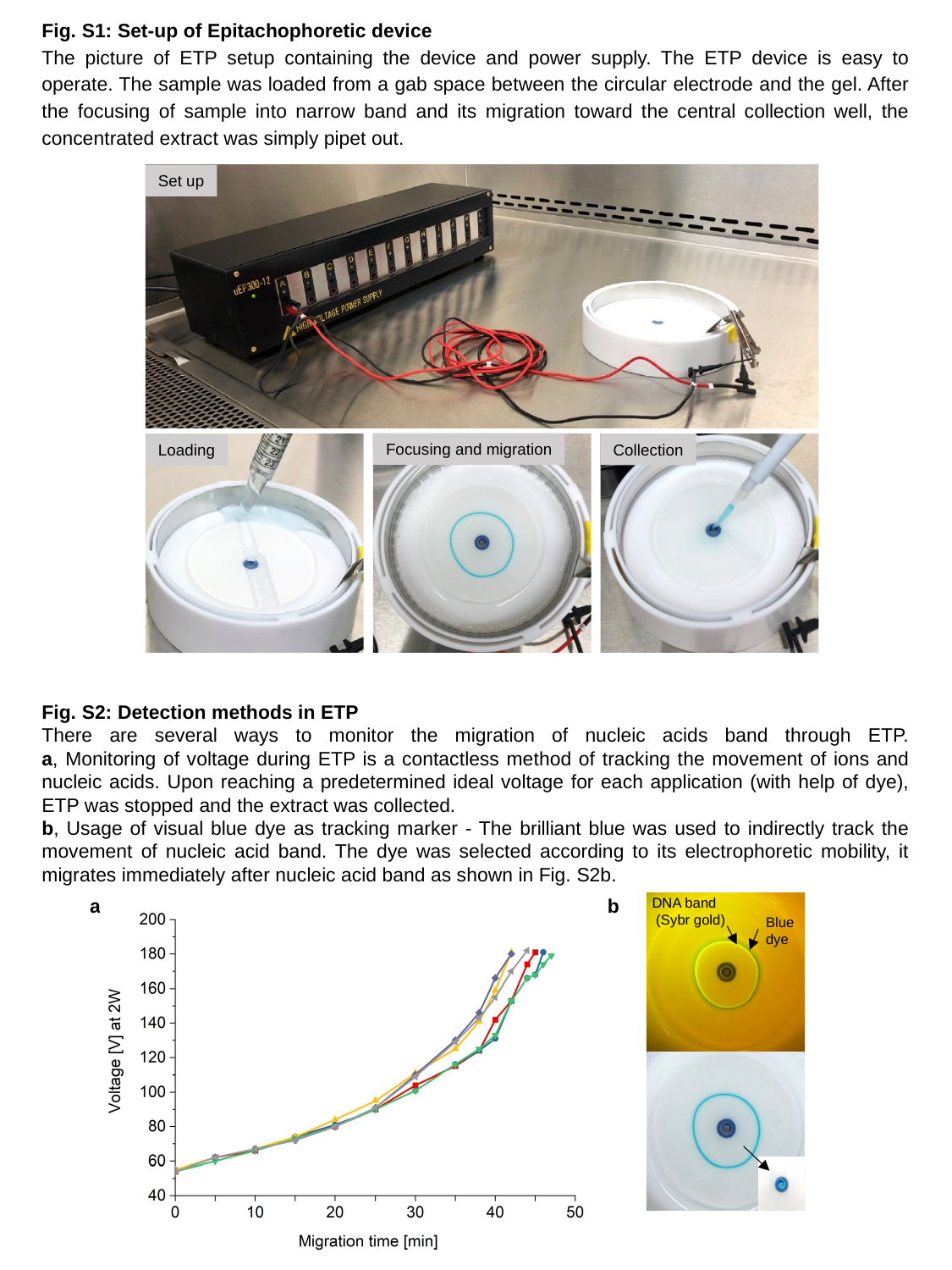

Fig. S1: Set-up of Epitachophoretic device
The picture of ETP setup containing the device and power supply. The ETP device is easy to operate. The sample was loaded from a gab space between the circular electrode and the gel. After the focusing of sample into narrow band and its migration toward the central collection well, the concentrated extract was simply pipet out.
Set up
Focusing and migration
Collection
Loading
Fig. S2: Detection methods in ETP
There are several ways to monitor the migration of nucleic acids band through ETP.
a, Monitoring of voltage during ETP is a contactless method of tracking the movement of ions and nucleic acids. Upon reaching a predetermined ideal voltage for each application (with help of dye), ETP was stopped and the extract was collected.
b, Usage of visual blue dye as tracking marker - The brilliant blue was used to indirectly track the movement of nucleic acid band. The dye was selected according to its electrophoretic mobility, it migrates immediately after nucleic acid band as shown in Fig. S2b.
DNA band
 (Sybr gold)
Blue
dye
a
b

## Slide 2
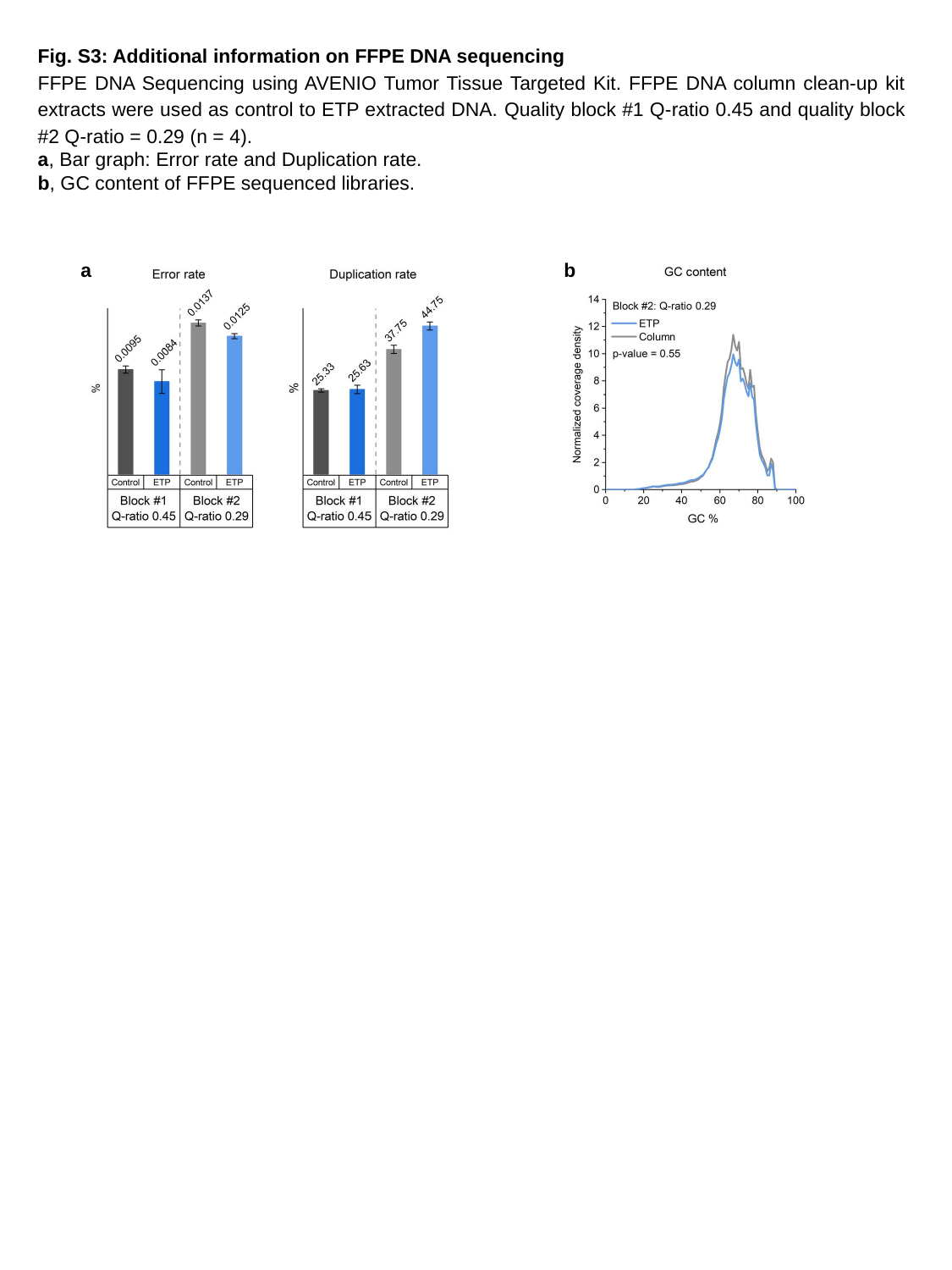

Fig. S3: Additional information on FFPE DNA sequencing
FFPE DNA Sequencing using AVENIO Tumor Tissue Targeted Kit. FFPE DNA column clean-up kit extracts were used as control to ETP extracted DNA. Quality block #1 Q-ratio 0.45 and quality block #2 Q-ratio = 0.29 (n = 4).
a, Bar graph: Error rate and Duplication rate.
b, GC content of FFPE sequenced libraries.
a
b

## Slide 3
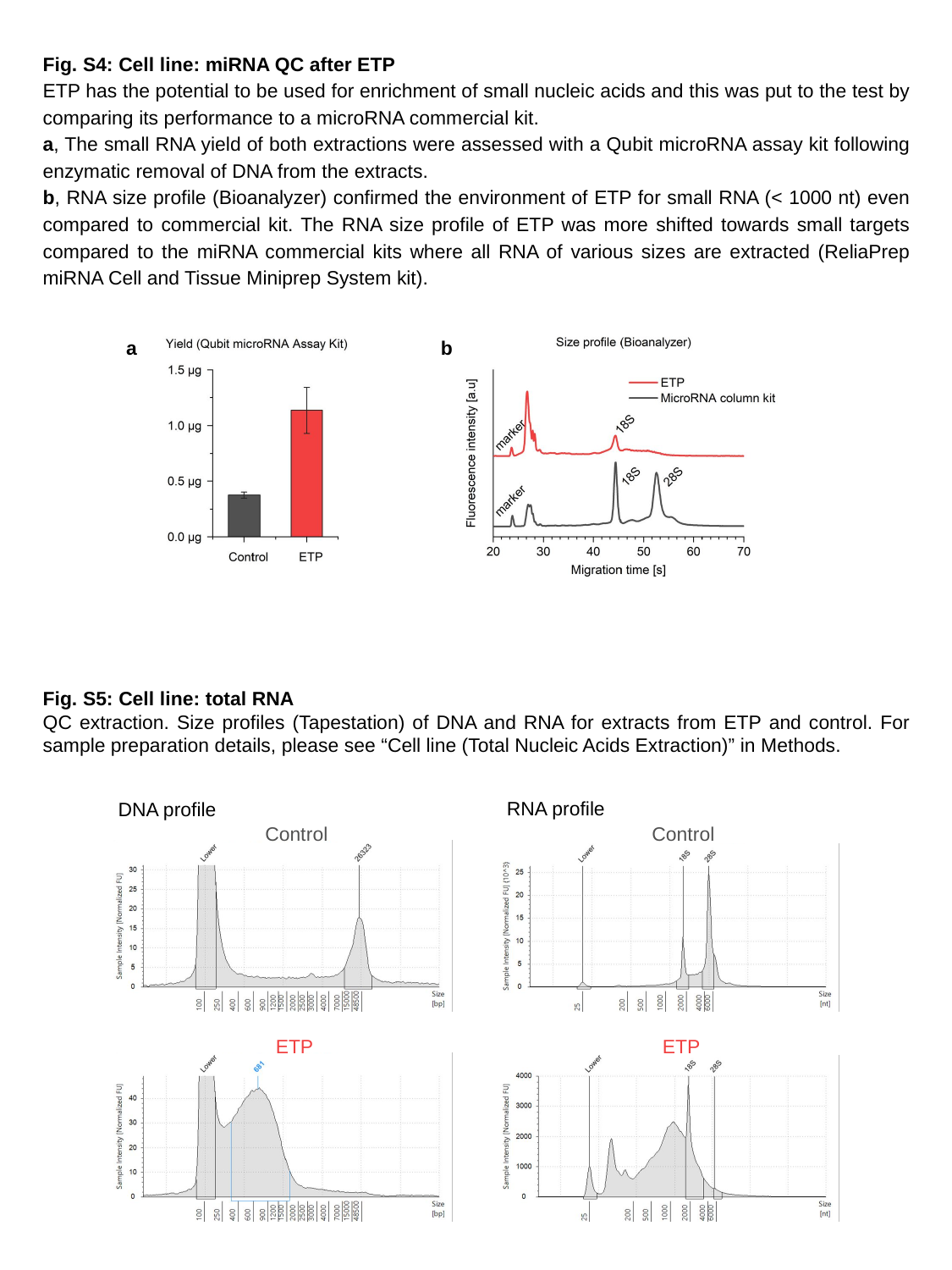

Fig. S4: Cell line: miRNA QC after ETP
ETP has the potential to be used for enrichment of small nucleic acids and this was put to the test by comparing its performance to a microRNA commercial kit.
a, The small RNA yield of both extractions were assessed with a Qubit microRNA assay kit following enzymatic removal of DNA from the extracts.
b, RNA size profile (Bioanalyzer) confirmed the environment of ETP for small RNA (< 1000 nt) even compared to commercial kit. The RNA size profile of ETP was more shifted towards small targets compared to the miRNA commercial kits where all RNA of various sizes are extracted (ReliaPrep miRNA Cell and Tissue Miniprep System kit).
a
b
Fig. S5: Cell line: total RNA
QC extraction. Size profiles (Tapestation) of DNA and RNA for extracts from ETP and control. For sample preparation details, please see “Cell line (Total Nucleic Acids Extraction)” in Methods.
RNA profile
DNA profile
Control
Control
ETP
ETP

## Slide 4
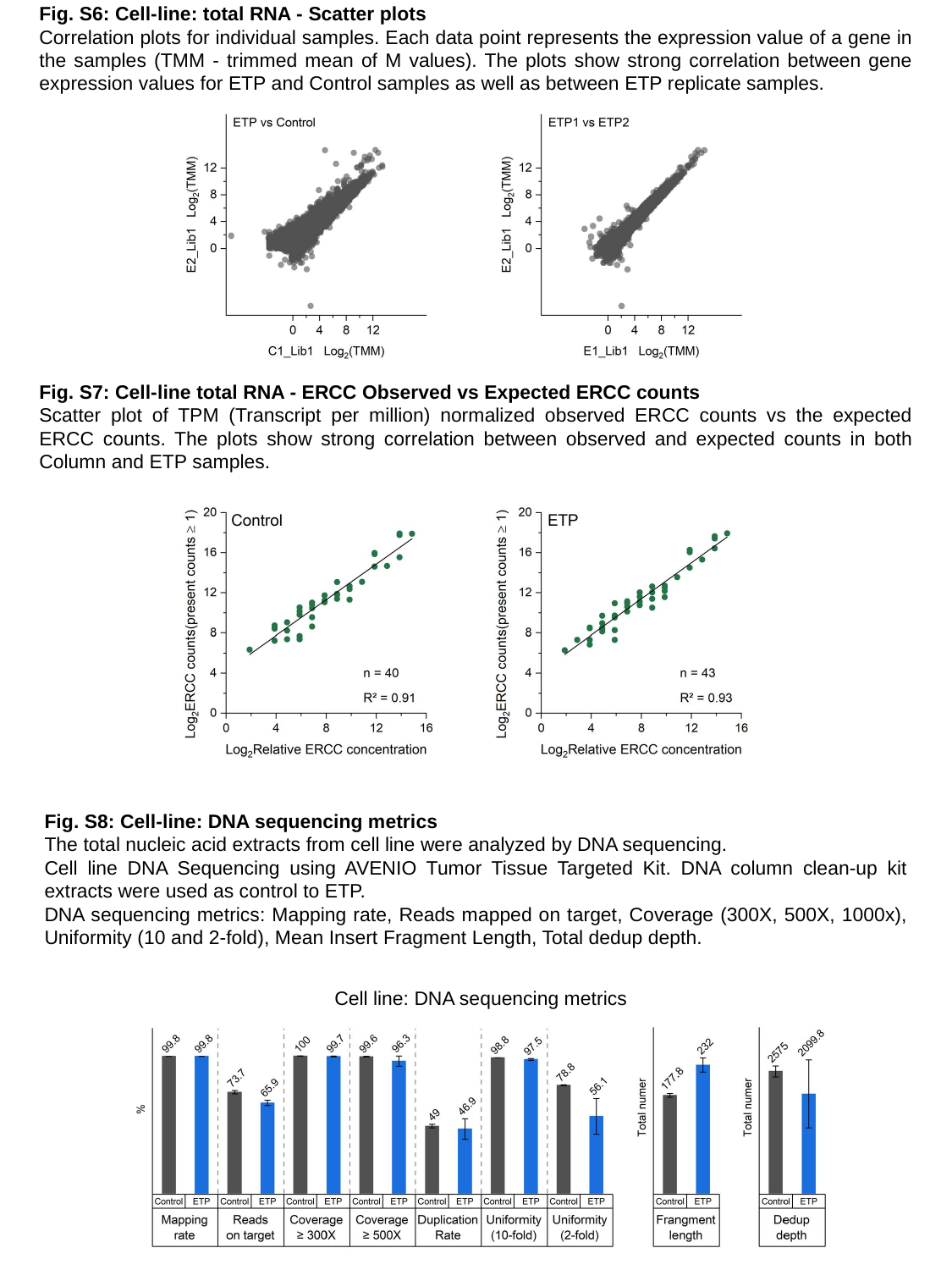

Fig. S6: Cell-line: total RNA - Scatter plots
Correlation plots for individual samples. Each data point represents the expression value of a gene in the samples (TMM - trimmed mean of M values). The plots show strong correlation between gene expression values for ETP and Control samples as well as between ETP replicate samples.
Fig. S7: Cell-line total RNA - ERCC Observed vs Expected ERCC counts
Scatter plot of TPM (Transcript per million) normalized observed ERCC counts vs the expected ERCC counts. The plots show strong correlation between observed and expected counts in both Column and ETP samples.
Fig. S8: Cell-line: DNA sequencing metrics
The total nucleic acid extracts from cell line were analyzed by DNA sequencing.
Cell line DNA Sequencing using AVENIO Tumor Tissue Targeted Kit. DNA column clean-up kit extracts were used as control to ETP.
DNA sequencing metrics: Mapping rate, Reads mapped on target, Coverage (300X, 500X, 1000x), Uniformity (10 and 2-fold), Mean Insert Fragment Length, Total dedup depth.
Cell line: DNA sequencing metrics

## Slide 5
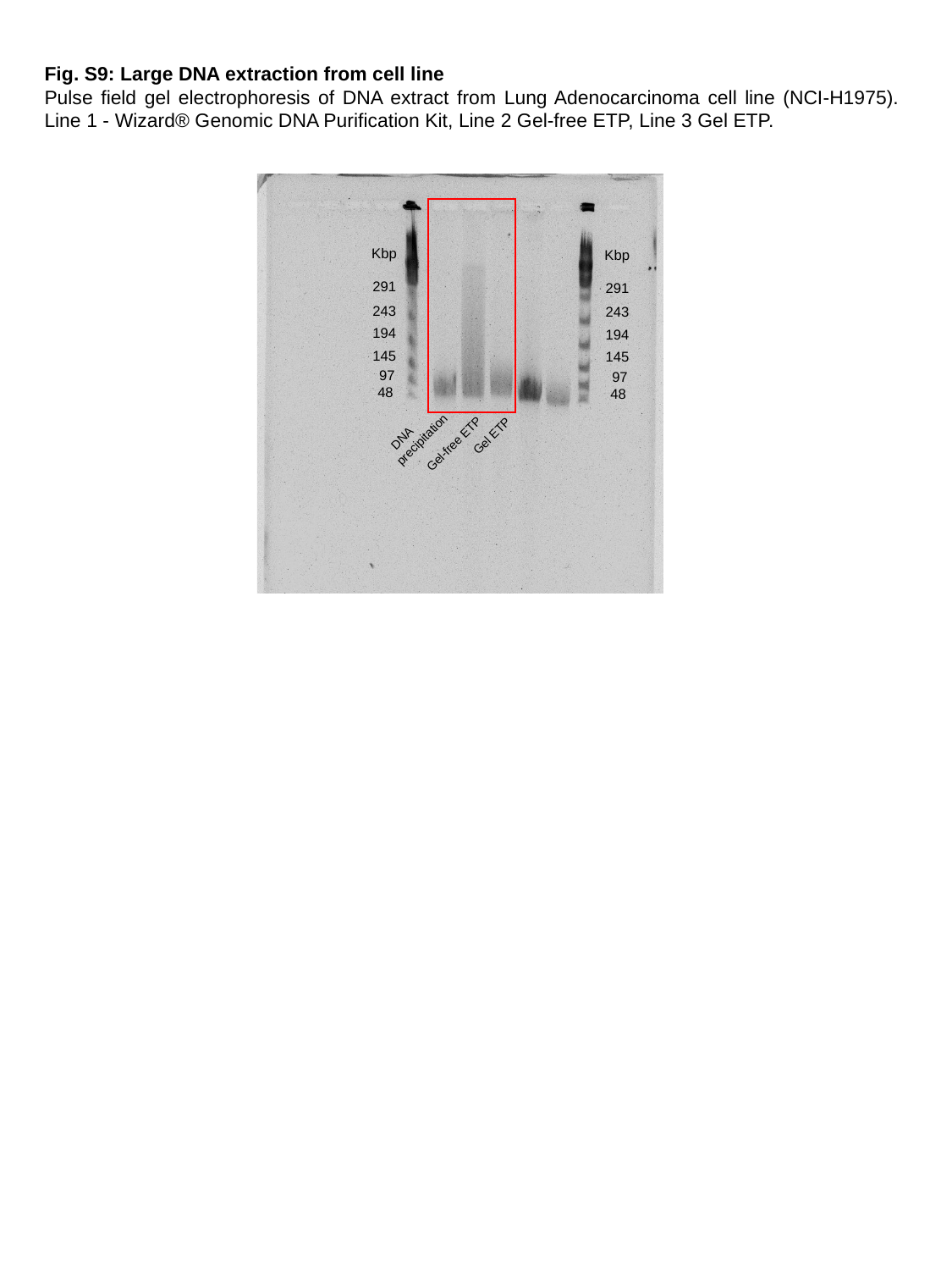

Fig. S9: Large DNA extraction from cell line
Pulse field gel electrophoresis of DNA extract from Lung Adenocarcinoma cell line (NCI-H1975). Line 1 - Wizard® Genomic DNA Purification Kit, Line 2 Gel-free ETP, Line 3 Gel ETP.
Kbp
Kbp
291
291
243
243
194
194
145
145
97
97
48
48
 DNA
precipitation
Gel ETP
Gel-free ETP
